# Flowers, Ferns, and Ward Decorations

**Published:** 1886-12-11

**Authors:** 


### FLOWERS, FERNS, AND WARD DECORATIONS.

[Continued from page 145.—Communications for this column should be addressed "Gardener," care of the Editor of THE HOSPITAL.]

MANY delicate flowers are no worse for having their stalks enveloped in damp moss or cotton-wool, to keep them cool and fresh. Camellias, gardenias, and such fragile things travel best with their stalks stuck through a piece of cardboard, to which they are made fast; the cardboard should have pieces of cork between it and the bottom of the box, and a sufficient space overhead, but the lid should not touch the contents. Some people run the stalks of these flowers through a skin of potato for the sake of dampness, but in no instance should the blooms be allowed to stir. Packed thus, they may be expected to come to hand quite unharmed, if they succeed in running the gauntlet of the train's jerkiness and the tender mercies bestowed on the unloading of the parcel-post! When first this mode of conveyance came into vogue I happened to witness the process in question at a busy station in the midland counties. When it was over, I felt like one who had been privileged to behold the transports of Vulcan more than usually playful with his thunderbolts or an earthquake joining issue with a lively volcano to accomplish the utter destruction of all things within reach of their united vengeance. My cries for mercy on the hampers and their possibly valuable contents were totally inaudible amid the crashing, shunting, steamwhistling, and bawling, which made existence hideous, and might have been heard by the clock on the town-hall. How those baskets held together passed the limits of human comprehension; and how the porter who stood on the platform to receive them escaped the fate of Samson amid the ruins of Gaza, is a fact I have never accounted for yet. Despite a liberal supply of Faith, Hope, and Charity, qualities absolutely indispensable in dealing with the parcel-post, I have never from that day to this committed to such means of transit anything more brittle than goloshes or a pattern bundle of Pryce Jones's choicest all-wool homespuns.

Before finishing "my say" for to-day, there is one little affair I would mention. Flowers have no wings, neither does their anatomy include the homelier addition of feet wherewith to conduct their own paces, and, consequently, those wards in a hospital which are nearest the clouds are frequently not so well supplied with them as those below-stairs. They who live closest to heaven must in this, as in other matters, forego many earthly delights, paying the penalty of their eminence in the loss of innumerable good things within the reach of less exalted mortals.

I let fall this hint for the benefit of all and sundry, trusting that some one will take it and *address by name* his or her contribution of flowers, from time to time, to such wards and individuals whose lines are cast several flights above the ground-floor. "I am too high up for many flowers to reach me," said a sister to me on one occasion; but I must not forget to say that the lady who uttered this gentle plaint presided over a ward, containing certainly the handsomest and best kept fern-cases and the largest and most interesting aquaria in all S——. I was just going to tell the name of the hospital, after managing, with the *utmost difficulty*, to keep it to myself throughout the whole length of this paper! Another day I will ask my readers to lend me their eyes again, when I may add something more on the subject of ward decorations.

